# Development of a model for predicting the severity of chronic obstructive pulmonary disease

**DOI:** 10.3389/fmed.2022.1073536

**Published:** 2022-12-16

**Authors:** Yu-Feng Gu, Long Chen, Rong Qiu, Shu-Hong Wang, Ping Chen

**Affiliations:** ^1^Department of Information, Suining Central Hospital, Suining, China; ^2^Department of Research Management, Suining Central Hospital, Suining, China; ^3^Department of Respiratory and Critical Care Medicine, Suining Central Hospital, Suining, China; ^4^Department of Nerve Central, Suining Central Hospital, Suining, China

**Keywords:** chronic obstructive pulmonary disease, prediction model, severity, retrospective study, biochemical and immunological

## Abstract

**Background:**

Several models have been developed to predict the severity and prognosis of chronic obstructive pulmonary disease (COPD). This study aimed to identify potential predictors and construct a prediction model for COPD severity using biochemical and immunological parameters.

**Methods:**

A total of 6,274 patients with COPD were recruited between July 2010 and July 2018. COPD severity was classified into mild, moderate, severe, and very severe based on the Global Initiative for Chronic Obstructive Lung Disease guidelines. A multivariate logistic regression model was constructed to identify predictors of COPD severity. The predictive ability of the model was assessed by measuring sensitivity, specificity, accuracy, and concordance.

**Results:**

Of 6,274 COPD patients, 2,644, 2,600, and 1,030 had mild/moderate, severe, and very severe disease, respectively. The factors that could distinguish between mild/moderate and severe cases were vascular disorders (OR: 1.44; *P* < 0.001), high-density lipoprotein (HDL) (OR: 1.83; *P* < 0.001), plasma fibrinogen (OR: 1.08; *P* = 0.002), fructosamine (OR: 1.12; *P* = 0.002), standard bicarbonate concentration (OR: 1.09; *P* < 0.001), partial pressure of carbon dioxide (OR: 1.09; *P* < 0.001), age (OR: 0.97; *P* < 0.001), eosinophil count (OR: 0.66; *P* = 0.042), lymphocyte ratio (OR: 0.97; *P* < 0.001), and apolipoprotein A1 (OR: 0.56; *P* = 0.003). The factors that could distinguish between mild/moderate and very severe cases were vascular disorders (OR: 1.59; *P* < 0.001), HDL (OR: 2.54; *P* < 0.001), plasma fibrinogen (OR: 1.10; *P* = 0.012), fructosamine (OR: 1.18; *P* = 0.001), partial pressure of oxygen (OR: 1.00; *P* = 0.007), plasma carbon dioxide concentration (OR: 1.01; *P* < 0.001), standard bicarbonate concentration (OR: 1.13; *P* < 0.001), partial pressure of carbon dioxide (OR: 1.16; *P* < 0.001), age (OR: 0.91; *P* < 0.001), sex (OR: 0.71; *P* = 0.010), allergic diseases (OR: 0.51; *P* = 0.009), eosinophil count (OR: 0.42; *P* = 0.014), lymphocyte ratio (OR: 0.93; *P* < 0.001), and apolipoprotein A1 (OR: 0.45; *P* = 0.005). The prediction model correctly predicted disease severity in 60.17% of patients, and kappa coefficient was 0.35 (95% CI: 0.33–0.37).

**Conclusion:**

This study developed a prediction model for COPD severity based on biochemical and immunological parameters, which should be validated in additional cohorts.

## Introduction

Chronic obstructive pulmonary disease (COPD) is characterized by persistent airflow obstruction to the lungs. This disease is not fully reversible and is a leading cause of adult morbidity worldwide (1–3). Approximately 251 million people had COPD worldwide in 2016, and this entity is expected to be the third leading cause of mortality by 2030 (4, 5). Although several treatment strategies for COPD are currently available, therapeutic effectiveness is limited because of disease heterogeneity, rapid progression, and complex pathophysiology (6, 7). Moreover, prognosis depends on disease severity, and risk stratification for COPD should be evaluated in patients whose disease was effectively managed.

Chronic obstructive pulmonary disease severity was classified by spirometry according to the Global Initiative for Chronic Obstructive Lung Disease (GOLD) guidelines, including forced expiratory volume in 1 s (FEV_1_) and FEV_1_/forced vital capacity (FVC) (8). The classification of COPD severity could affects prognosis of COPD and appropriate treatment strategies should be applied (9–11). COPD patients have multiple comorbidities and changes in the immunobiochemical index, which should be included in the GOLD staging guidelines (12). However, previous studies developed models to predict mortality and disease exacerbation in patients with stable COPD, but not prediction models for COPD severity (13–15).

A prediction model for COPD severity was developed and validated using a claims-based algorithm. The final model included age, sex, comorbidities, COPD-related resource utilization, and all-cause healthcare utilization (16). The model correctly predicted disease severity in 62.7% of cases in the validation set. However, the model did not include biochemical and immunological parameters. This real-world study constructed a prediction model for COPD severity and identified influencing factors.

## Materials and methods

### Data source

This retrospective cohort study recruited 6,274 patients who presented to our hospital between July 2010 and July 2018. The inclusion criteria were adult patients with COPD and a history of chest tightness, chest pain, and cardiac discomfort of varying durations. The COPD was defined as post-bronchodilator fixed ratio of FEV_1_/FVC < 70% according to GOLD criteria (8). The exclusion criteria were patients with incomplete clinical data, severe infections, chronic liver and renal dysfunction, and immune dysfunction. The Research Ethics Committee of Suining Central Hospital approved this study on 26 January 2021 (No.: 2018-19). The need for informed consent was waived because of the retrospective design of the study.

### Data collection

Demographic and clinical data were extracted from electronic medical records, including age, sex, diabetes mellitus, vascular disorders, psychiatric disorders, infections, allergic diseases, hypertension, tumors, gastrointestinal diseases, and osteoarticular diseases. In addition, data on the following biochemical and immunological parameters were collected: platelet-large cell ratio (P-LCR), platelet count, eosinophil count, mean platelet volume (MPV), eosinophil ratio, basophilic granulocytes, platelet distribution width (PDW), lymphocyte count, lymphocyte ratio, basophil ratio, low-density lipoprotein (LDL)/high-density lipoprotein (HDL) ratio, HDL, albumin/globulin ratio, serum iron, indirect bilirubin, direct bilirubin/total bilirubin ratio, globulin, magnesium, LDL, erythrocyte sedimentation rate (ESR), apolipoprotein A1, lipoprotein (a), carcinoembryonic antigen (CEA), plasma fibrinogen, prothrombin time (PT), PT activity, plasma thrombin time, fructosamine, myoglobin, cystatin C, serum procalcitonin, partial pressure of oxygen (pO_2_), plasma carbon dioxide concentration, bicarbonate concentration, standard bicarbonate concentration, extracellular fluid volume depletion, partial pressure of carbon dioxide (pCO_2_), base excess, percentage and absolute number of granulocytes, high-sensitivity C-reactive protein (hsCRP), and D-dimer.

### COPD severity

The severity of COPD was classified as mild, moderate, severe, and very severe, according to the GOLD guidelines (8). Mild, moderate, severe, and very severe COPD was defined as measured/predicted FEV_1_ after bronchodilator inhalation ≥80, 50–79, 30–49, and <30%, respectively. These categories were combined into three (mild/moderate, severe, and very severe) to standardize the number of patients in each group.

### Statistical analysis

The data were presented as mean ± standard deviation for normally distributed continuous variables and as medians (interquartile range) for non-normally distributed continuous variables. Categorical variables were expressed as numbers and percentages. Differences between groups were assessed using analysis of variance, the Kruskal–Wallis test, and the chi-square test. Potential predictors of COPD severity were identified by logistic regression, and COPD severity was considered the dependent variable. Covariates with a *P* < 0.05 were included in the multivariate regression model. The final set of predictors was determined using stepwise selection at a threshold of *P* < 0.05. The multivariable model was used to predict the probability of each patient being assigned to each COPD severity. Model performance was assessed by calculating sensitivity, specificity, and accuracy. The agreement between predicted and actual values was assessed using Cohen’s kappa test. All statistical tests were two-sided, and *P*-values of less than 0.05 were considered statistically significant. All statistical analyses were conducted using SPSS Statistics 19.0 for Windows (IBM Corp., Armonk, NY, USA).

## Results

### Baseline characteristics

A total of 2,644, 2,600, and 1,030 patients had mild/moderate, severe, and very severe COPD. There were significant differences among three groups for mostly variables, including age, sex, vascular disorders, psychiatric disorders, infections, allergic diseases, hypertension, tumors, gastrointestinal diseases, osteoarticular diseases, P-LCR, platelet count, eosinophil count, MPV, eosinophil ratio, basophilic granulocytes, PDW, lymphocyte count, lymphocyte ratio, basophil ratio, LDL/HDL ratio, HDL, albumin/globulin ratio, serum iron, indirect bilirubin, direct bilirubin/total bilirubin ratio, globulin, magnesium, ESR, CEA, plasma fibrinogen, PT, PT activity, plasma thrombin time, cystatin C, serum procalcitonin, pO_2_, plasma carbon dioxide concentration, bicarbonate concentration, standard bicarbonate concentration, extracellular fluid volume depletion, pCO_2_, base excess, and hsCRP. However, there were no significant differences in diabetes mellitus prevalence, LDL, apolipoprotein A1, lipoprotein (a), fructosamine, myoglobin, percentage of granulocytes, absolute number of granulocytes, and D-dimer concentration. The details characteristics of the cohorts among three groups are shown in Table 1.

**TABLE 1:** Baseline characteristics of included patients.

Variable	Overall (*n* = 6,274)	COPD			*P*-value
		**Mild/Moderate (*n* = 2,644)**	**Severe (*n* = 2,600)**	**Very severe (*n* = 1,030)**	
Age	70.00 (64.00, 77.00)	72.00 (65.00, 78.00)	71.00 (64.00, 77.00)	66.00 (60.25, 72.00)^b,c^	<0.001
Sex (female)	1858 (29.61)	803 (30.37)	804 (30.92)	251 (24.37)^b,c^	<0.001
DM	164 (2.61)	75 (2.84)	61 (2.35)	28 (2.72)	0.524
Vascular disorder	1691 (26.95)	607 (22.96)	752 (28.92)^a^	332 (32.23)^b^	<0.001
Psychiatric disorder	14 (0.22)	2 (0.08)	7 (0.27)	5 (0.49)^b^	0.036
Infection	1355 (21.60)	478 (18.08)	595 (22.88)^a^	282 (27.38)^b,c^	<0.001
Allergic disease	439 (7.00)	225 (8.51)	169 (6.50)	45 (4.37)^b^	<0.001
Hypertension	400 (6.38)	207 (7.83)	146 (5.62)	47 (4.56)^b^	<0.001
Tumor	123 (1.96)	72 (2.72)	43 (1.65)	8 (0.78)^b^	<0.001
Gastrointestinal disease	415 (6.61)	191 (7.22)	174 (6.69)	50 (4.85)^b^	0.034
Osteoarticular disease	87 (1.39)	48 (1.82)	34 (1.31)	5 (0.49)^b^	0.007
Platelet-large cell ratio	37.05 (30.45, 44.30)	37.85 (30.80, 45.55)	36.80 (30.10, 43.60)^a^	36.20 (30.09, 43.21)^b,c^	<0.001
Thrombocytocrit	0.21 (0.17, 0.26)	0.21 (0.17, 0.26)	0.21 (0.17, 0.26)	0.20 (0.16, 0.24)^b^	<0.001
Eosinophils	0.10 (0.05, 0.19)	0.11 (0.06, 0.20)	0.10 (0.05, 0.19)	0.09 (0.04, 0.17)^b^	<0.001
Mean platelet volume	11.55 (10.70, 12.50)	11.62 (10.78, 12.69)	11.50 (10.65, 12.40)^a^	11.45 (10.65, 12.32)^b^	<0.001
Eosinophil ratio	1.50 (0.65, 2.85)	1.70 (0.80, 3.10)	1.50 (0.60, 2.80)^a^	1.14 (0.45, 2.48)^b,c^	<0.001
Basophilic granulocyte	0.02 (0.01, 0.03)	0.02 (0.01, 0.03)	0.02 (0.01, 0.03)^a^	0.02 (0.01, 0.03)^b,c^	<0.001
Platelet distribution width	14.93 (12.86, 17.20)	15.05 (12.90, 17.39)	14.80 (12.78, 17.00)^a^	14.94 (13.00, 16.99)^b^	0.038
Lymphocytes	1.19 (0.88, 1.57)	1.27 (0.94, 1.68)	1.16 (0.86, 1.51)^a^	1.06 (0.76, 1.42)^b,c^	<0.001
Lymphocyte ratio	16.70 (11.80, 22.70)	18.55 (13.35, 24.90)	16.09 (11.40, 21.60)^a^	14.28 (9.98, 19.20)^b,c^	<0.001
Basophils ratio	0.25 (0.15, 0.40)	0.30 (0.15, 0.45)	0.25 (0.12, 0.40)^a^	0.20 (0.12, 0.36)^b,c^	<0.001
LDL/HDL ratio	1.63 (1.22, 2.15)	1.70 (1.29, 2.19)	1.59 (1.18, 2.12)^a^	1.53 (1.13, 2.03)^b^	<0.001
HDL	1.42 (1.14, 1.73)	1.35 (1.09, 1.64)	1.44 (1.16, 1.75)^a^	1.50 (1.19, 1.88)^b,c^	<0.001
Albumin/globulin ratio	1.30 (1.18, 1.50)	1.30 (1.20, 1.50)	1.30 (1.15, 1.50)	1.35 (1.20, 1.54)^b,c^	0.012
Serum iron	9.90 (5.80, 14.90)	10.60 (6.20, 15.50)	9.53 (5.60, 14.30)^a^	9.40 (5.60, 14.50)^b^	<0.001
Indirect bilirubin	5.80 (4.10, 8.10)	6.00 (4.20, 8.20)	5.70 (4.09, 7.90)^a^	5.80 (3.85, 8.00)	0.044
Direct bilirubin/total bilirubin ratio	0.37 (0.31, 0.45)	0.36 (0.30, 0.44)	0.37 (0.31, 0.45)^a^	0.38 (0.32, 0.47)^b,c^	<0.001
Globulin	29.10 (25.80, 32.60)	29.40 (26.30, 32.80)	28.90 (25.70, 32.50)^a^	28.30 (24.85, 31.95)^b,c^	<0.001
Magnesium	0.85 (0.79, 0.92)	0.86 (0.80, 0.92)	0.85 (0.79, 0.91)^a^	0.84 (0.76, 0.91)^b,c^	<0.001
LDL	2.28 (1.78, 2.87)	2.30 (1.82, 2.84)	2.26 (1.78, 2.90)	2.25 (1.72, 2.89)	0.613
Erythrocyte sedimentation rate	21.00 (9.00, 43.00)	23.00 (11.00, 44.75)	22.31 (10.00, 45.12)	14.00 (5.00, 31.50)^b,c^	<0.001
Apolipoprotein A1	1.37 (1.16, 1.61)	1.36 (1.16, 1.59)	1.39 (1.16, 1.63)	1.38 (1.15, 1.63)	0.450
Lipoprotein (a)	87.00 (30.00, 190.01)	90.00 (29.90, 186.00)	81.00 (32.05, 189.00)	91.05 (26.52, 204.00)	0.853
Carcino-embryonic antigen	2.90 (1.90, 4.30)	2.70 (1.80, 4.10)	2.90 (1.92, 4.24)^a^	3.40 (2.28, 4.82)^b,c^	<0.001
Plasma fibrinogen	4.35 (3.44, 5.58)	4.27 (3.41, 5.49)	4.47 (3.53, 5.68)^a^	4.30 (3.30, 5.56)^c^	<0.001
Prothrombin time	13.30 (12.60, 14.10)	13.30 (12.60, 14.00)	13.30 (12.60, 14.10)	13.40 (12.70, 14.40)^b,c^	0.006
Prothrombin time activity	98.00 (87.00, 110.00)	99.00 (88.00, 110.00)	98.00 (87.00, 110.00)	97.00 (83.25, 109.00)^b,c^	0.002
Plasma thrombin time	16.80 (15.90, 18.00)	16.80 (15.90, 17.80)	16.80 (15.80, 18.00)	17.10 (16.10, 18.38)^b,c^	<0.001
Fructosamine	2.00 (1.70, 225.00)	2.00 (1.70, 223.00)	2.00 (1.70, 226.00)	2.00(1.70,228.00)	0.501
Myoglobin	43.17 (30.63, 65.41)	43.00 (30.69, 65.01)	44.00 (31.15, 67.18)	42.08(29.21,62.04)	0.144
Cystatin C	1.01 (0.87, 1.20)	1.03 (0.89, 1.23)	1.01 (0.86, 1.20)^a^	0.98(0.83,1.12)^b,c^	<0.001
Serum procalcitonin	0.08 (0.05, 0.17)	0.08 (0.05, 0.16)	0.09 (0.06, 0.18)^a^	0.09(0.06,0.18)^b^	<0.001
Oxygen partial pressure	93.97 (74.00, 127.99)	94.00 (74.70, 131.30)	94.10 (74.00, 126.65)	92.40(71.46,123.96)^b,c^	0.026
Plasma carbon dioxide concentration	46.00 (30.30, 60.75)	37.30 (28.00, 56.90)	45.50 (31.20, 62.20)^a^	57.10(37.71,71.61)^b,c^	<0.001
Bicarbonate concentration	27.90 (25.20, 31.64)	25.90 (24.00, 28.40)	28.75 (26.00, 32.35)^a^	32.75(29.10,36.20)^b,c^	<0.001
Bicarbonate standard value	26.50 (24.60, 28.80)	25.40 (23.80, 27.10)	27.00 (25.10, 29.15)^a^	28.80(26.64,31.15)^b,c^	<0.001
Residual base in extracellular fluid	3.80 (1.30, 7.30)	1.90 (0.10, 4.20)	4.55 (2.00, 7.70)^a^	7.79(4.50,10.90)^b,c^	<0.001
Partial pressure of carbon dioxide	46.00 (41.00, 53.86)	42.00 (38.10, 46.05)	47.60 (42.50, 55.00)^a^	57.36(49.00,65.75)^b,c^	<0.001
Base excess	3.10 (1.00, 5.80)	1.60 (0.10, 3.50)	3.60 (1.50, 6.10)^a^	5.85(3.30,8.50)^b,c^	<0.001
Percentage of naive granulocytes	0.40 (0.25, 0.72)	0.40 (0.25, 0.75)	0.40 (0.25, 0.71)	0.40(0.28,0.65)	0.385
Absolute value of naive granulocytes	0.03 (0.02, 0.06)	0.03 (0.01, 0.06)	0.03 (0.01, 0.06)	0.03(0.02,0.06)	0.880
High sensitivity C-reactive protein	14.86 (5.90, 37.51)	14.33 (5.60, 36.15)	15.84 (6.11, 40.26)^a^	13.80(5.68,34.33)^c^	0.008
D-dimer	1.18 (0.62, 1.94)	1.15 (0.61, 1.85)	1.16 (0.62, 1.99)	1.29(0.71,2.07)	0.059

^a^*P*-value for severe versus mild/moderate COPD < 0.05; ^b^*P*-value for very severe versus mild/moderate COPD < 0.05; ^c^*P*-value for very severe versus severe COPD < 0.05.

### Univariate analysis of COPD severity

The results of the univariate analysis are shown in Table 2. Vascular disorders, infections, HDL, direct bilirubin/total bilirubin ratio, plasma fibrinogen, plasma carbon dioxide concentration, bicarbonate concentration, standard bicarbonate concentration, extracellular fluid volume depletion, pCO_2_, base excess, and hsCRP were significantly associated with an increased risk of severe COPD. In contrast, age, allergic diseases, hypertension, tumors, P-LCR, eosinophil count, MPV, eosinophil ratio, PDW, lymphocyte count, lymphocyte ratio, basophil ratio, LDL/HDL ratio, globulin, and magnesium were significantly associated with a reduced risk of severe COPD.

**TABLE 2:** Univariable multinomial logistic regression for the severity of chronic obstructive pulmonary disease (COPD).

Variable	Severe vs. mild/moderate				Very severe vs. mild/moderate			
	**β**	**OR and 95% CI**	**χ^2^**	***P*** **-value**	**β**	**OR and 95% CI**	**χ^2^**	***P*** **-value**
Age	–0.010	0.99 (0.98∼1.00)	11.038	0.001	–0.06	0.94 (0.93∼0.95)	234.110	<0.001
Sex (female vs. male)	0.026	1.03 (0.91∼1.15)	0.188	0.664	–0.303	0.74 (0.63∼0.87)	12.990	<0.001
DM (yes vs. no)	–0.195	0.82 (0.58∼1.16)	1.245	0.265	–0.044	0.96 (0.62∼1.49)	0.038	0.846
Vascular disorder (yes vs. no)	0.312	1.37 (1.21∼1.55)	24.217	<0.001	0.468	1.60 (1.36∼1.87)	33.221	<0.001
Psychiatric disorder (yes vs. no)	1.271	3.57 (0.74∼17.18)	2.512	0.113	1.863	6.44 (1.25∼33.27)	4.949	0.026
Infection (yes vs. no)	0.296	1.34 (1.18∼1.54)	18.541	<0.001	0.536	1.71 (1.44∼2.02)	38.588	<0.001
Allergic disease (yes vs. no)	–0.291	0.75 (0.61∼0.92)	7.578	0.006	–0.711	0.49 (0.35∼0.68)	17.991	<0.001
Hypertension (yes vs. no)	–0.356	0.70 (0.56∼0.87)	10.139	0.001	–0.574	0.56 (0.41∼0.78)	11.978	0.001
Tumor (yes vs. no)	–0.510	0.60 (0.41∼0.88)	6.848	0.009	–1.274	0.28 (0.13∼0.58)	11.574	0.001
Gastrointestinal disease (yes vs. no)	–0.082	0.92 (0.74∼1.14)	0.571	0.450	–0.422	0.66 (0.48∼0.90)	6.685	0.010
Osteoarticular disease (yes vs. no)	–0.333	0.72 (0.46∼1.12)	2.176	0.140	–1.331	0.26 (0.10∼0.67)	7.984	0.005
Platelet-large cell ratio	–0.013	0.99 (0.98∼0.99)	19.330	<0.001	–0.015	0.98 (0.98∼0.99)	15.005	<0.001
Thrombocytocrit	–0.368	0.69 (0.32∼1.49)	0.886	0.347	–2.651	0.07 (0.02∼0.21)	23.023	<0.001
Eosinophils	–0.588	0.56 (0.41∼0.75)	14.581	<0.001	–1.107	0.33 (0.20∼0.54)	20.089	<0.001
Mean platelet volume	–0.100	0.90 (0.87∼0.95)	20.251	<0.001	–0.115	0.89 (0.84∼0.94)	15.235	<0.001
Eosinophil ratio	–0.050	0.95 (0.93∼0.97)	20.854	<0.001	–0.118	0.89 (0.86∼0.92)	41.232	<0.001
Basophilic granulocyte	–3.057	0.05 (0.00∼1.03)	3.757	0.053	–8.272	0.00 (0.00∼0.02)	13.518	<0.001
Platelet distribution width	–0.025	0.98 (0.96∼0.99)	7.737	0.005	–0.012	0.99 (0.96∼1.01)	1.064	0.302
Lymphocytes	–0.436	0.65 (0.59∼0.71)	73.584	<0.001	–0.755	0.47 (0.41∼0.54)	104.705	<0.001
Lymphocyte ratio	–0.043	0.96 (0.95∼0.96)	141.605	<0.001	–0.077	0.93 (0.92∼0.94)	212.419	<0.001
Basophils ratio	–0.428	0.65 (0.53∼0.81)	15.211	<0.001	–1.091	0.34 (0.24∼0.47)	43.082	<0.001
LDL/HDL ratio	–0.066	0.94 (0.88∼0.99)	4.711	0.030	–0.254	0.78 (0.70∼0.86)	25.384	<0.001
HDL	0.377	1.46 (1.29∼1.64)	38.658	<0.001	0.635	1.89 (1.62∼2.19)	69.213	<0.001
Albumin/globulin ratio	0.036	1.04 (0.88∼1.23)	0.173	0.677	0.233	1.26 (1.04∼1.54)	5.484	0.019
Serum iron*	–0.249	0.78 (0.49∼1.25)	1.054	0.305	–0.518	0.60 (0.25∼1.43)	1.337	0.248
Indirect bilirubin*	–0.105	0.90 (0.35∼2.31)	0.047	0.828	–0.269	0.76 (0.19∼3.14)	0.139	0.710
Direct bilirubin/total bilirubin ratio	0.513	1.67 (1.09∼2.56)	5.568	0.018	0.894	2.44 (1.45∼4.12)	11.315	0.001
Globulin	–0.015	0.98 (0.98∼0.99)	9.069	0.003	–0.040	0.96 (0.95∼0.97)	32.174	<0.001
Magnesium	–0.987	0.37 (0.23∼0.60)	16.125	<0.001	–2.091	0.12 (0.06∼0.25)	35.818	<0.001
LDL	0.023	1.02 (0.96∼1.09)	0.466	0.495	0.009	1.01 (0.92∼1.10)	0.043	0.836
Erythrocyte sedimentation rate	–0.001	1.00 (1.00∼1.00)	0.556	0.456	–0.017	0.98 (0.98∼0.99)	63.475	<0.001
Apolipoprotein A1	0.032	1.03 (0.89∼1.20)	0.173	0.677	0.036	1.04 (0.85∼1.27)	0.119	0.730
Lipoprotein (a)*	–0.004	1.00 (0.97∼1.03)	0.061	0.804	0.023	1.02 (0.99∼1.06)	1.435	0.231
Carcino-embryonic antigen*	–0.125	0.88 (0.69∼1.12)	1.036	0.309	–0.111	0.90 (0.64∼1.26)	0.408	0.523
Plasma fibrinogen	0.070	1.07 (1.03∼1.11)	14.806	<0.001	0.013	1.01 (0.97∼1.06)	0.299	0.585
Prothrombin time	0.027	1.03 (0.99∼1.07)	1.908	0.167	0.088	1.09 (1.04∼1.14)	14.730	<0.001
Prothrombin time activity	–0.002	1.00 (1.00∼1.00)	1.000	0.317	–0.008	0.99 (0.99∼1.00)	14.190	<0.001
Plasma thrombin time	0.015	1.02 (0.99∼1.05)	0.980	0.322	0.082	1.09 (1.05∼1.12)	21.153	<0.001
Fructosamine*	0.039	1.04 (0.99∼1.09)	2.545	0.111	0.072	1.08 (1.01∼1.15)	5.025	0.025
Myoglobin*	0.045	1.05 (0.92∼1.19)	0.480	0.489	–0.054	0.95 (0.79∼1.14)	0.332	0.565
Cystatin C	–0.116	0.89 (0.77∼1.03)	2.457	0.117	–0.865	0.42 (0.32∼0.55)	41.269	<0.001
Serum procalcitonin	–0.013	0.99 (0.96∼1.01)	0.932	0.334	–0.017	0.98 (0.94∼1.02)	0.655	0.418
Oxygen partial pressure	–0.001	1.00 (1.00∼1.00)	3.154	0.076	–0.003	1.00 (1.00∼1.00)	8.722	0.003
Plasma carbon dioxide concentration	0.018	1.02 (1.01∼1.02)	101.432	<0.001	0.044	1.05 (1.04∼1.05)	371.329	<0.001
Bicarbonate concentration	0.180	1.20 (1.18∼1.22)	490.15	<0.001	0.308	1.36 (1.33∼1.39)	921.163	<0.001
Bicarbonate standard value	0.207	1.23 (1.20∼1.26)	339.624	<0.001	0.355	1.43 (1.39∼1.47)	641.054	<0.001
Residual base in extracellular fluid	0.182	1.20 (1.18∼1.22)	397.406	<0.001	0.304	1.35 (1.33∼1.38)	749.258	<0.001
Partial pressure of carbon dioxide	0.094	1.10 (1.09∼1.11)	518.842	<0.001	0.162	1.18 (1.16∼1.19)	1024.067	<0.001
Base excess	0.202	1.22 (1.20∼1.25)	340.980	<0.001	0.337	1.40 (1.37∼1.44)	647.110	<0.001
Percentage of naive granulocytes	–0.005	0.99 (0.93∼1.07)	0.019	0.890	–0.119	0.89 (0.79∼0.99)	4.429	0.035
Absolute value of naive granulocytes	0.211	1.24 (0.63∼2.41)	0.386	0.534	–0.502	0.61 (0.23∼1.61)	1.011	0.315
High sensitivity C-reactive protein*	0.200	1.22 (1.02∼1.47)	4.486	0.034	–0.106	0.90 (0.69∼1.17)	0.639	0.424
D-dimer	0.020	1.02 (0.99∼1.05)	1.601	0.206	0.031	1.03 (1.00∼1.07)	2.897	0.089

*Presented as absolute value/100.

Vascular and psychiatric disorders, infections, HDL, albumin/globulin ratio, direct bilirubin/total bilirubin ratio, PT, plasma thrombin time, fructosamine, plasma carbon dioxide concentration, bicarbonate concentration, standard bicarbonate concentration, extracellular fluid volume depletion, pCO_2_, and base excess were associated with an increased risk of very severe COPD. In turn, sex, allergic diseases, hypertension, tumors, gastrointestinal and osteoarticular diseases, P-LCR, platelet count, eosinophil count, MPV, eosinophil ratio, basophilic granulocytes, lymphocyte count, lymphocyte ratio, basophil ratio, LDL/HDL ratio, globulin, magnesium, ESR, PT activity, cystatin C, and the percentage of granulocytes were associated with a reduced risk of very severe COPD.

### Multivariate analysis of COPD severity

The results of the multivariate analysis are shown in Table 3. After adjusting for potential confounders, vascular disorders (OR: 1.44; 95% CI: 1.21–1.71; *P* < 0.001), HDL (OR: 1.83; 95% CI: 1.38–2.43; *P* < 0.001), plasma fibrinogen (OR: 1.08; 95% CI: 1.03–1.14; *P* = 0.002), fructosamine (OR: 1.12; 95% CI: 1.04–1.20; *P* = 0.002), standard bicarbonate concentration (OR: 1.09; 95% CI: 1.05–1.13; *P* < 0.001), and pCO_2_ (OR: 1.09; 95% CI: 1.07–1.10; *P* < 0.001) were significantly associated with an increased risk of severe COPD. In contrast, age (OR: 0.97; 95% CI: 0.96–0.98; *P* < 0.001), eosinophil count (OR: 0.66; 95% CI: 0.44–0.98; *P* = 0.042), lymphocyte ratio (OR: 0.97; 95% CI: 0.96–0.98; *P* < 0.001), and apolipoprotein A1 (OR: 0.56; 95% CI: 0.38–0.82; *P* = 0.003) were significantly associated with a reduced risk of severe COPD. Vascular disorders (OR: 1.59; 95% CI: 1.24–2.03; *P* < 0.001), HDL (OR: 2.54; 95% CI: 1.70–3.79; *P* < 0.001), plasma fibrinogen (OR: 1.10; 95% CI: 1.02–1.18; *P* = 0.012), fructosamine (OR: 1.18; 95% CI: 1.07–1.31; *P* = 0.001), pO_2_ (OR: 1.00; 95% CI: 1.00–1.01; *P* = 0.007), plasma carbon dioxide concentration (OR: 1.01; 95% CI: 1.01–1.02; *P* < 0.001), standard bicarbonate concentration (OR: 1.13; 95% CI: 1.07–1.18; *P* < 0.001), and pCO_2_ (OR: 1.16; 95% CI: 1.14–1.18; *P* < 0.001) were significantly associated with an increased risk of very severe COPD. In turn, age (OR: 0.91; 95% CI: 0.90–0.92; *P* < 0.001), sex (OR: 0.71; 95% CI: 0.55–0.92; *P* = 0.010), allergic diseases (OR: 0.51; 95% CI: 0.31–0.84; *P* = 0.009), eosinophil count (OR: 0.42; 95% CI: 0.21–0.84; *P* = 0.014), lymphocyte ratio (OR: 0.93; 95% CI: 0.91–0.95; *P* < 0.001), and apolipoprotein A1 (OR: 0.45; 95% CI: 0.26–0.79; *P* = 0.005) were associated with a reduced risk of very severe COPD.

**TABLE 3:** Multivariable multinomial logistic regression for the severity of chronic obstructive pulmonary disease (COPD).

Variable	Severe vs. mild/moderate				Very severe vs. Mild/moderate			
	**β**	**OR and 95% CI**	**χ^2^**	*P* **-value**	**β**	**OR and 95% CI**	**χ^2^**	***P*** **-value**
Age	–0.028	0.97 (0.96∼0.98)	40.577	<0.001	–0.098	0.91 (0.90∼0.92)	231.639	<0.001
Sex (female vs. male)	0.050	1.05 (0.89∼1.25)	0.325	0.568	–0.337	0.71 (0.55∼0.92)	6.640	0.010
Vascular disorder (yes vs. no)	0.365	1.44 (1.21∼1.71)	17.031	<0.001	0.463	1.59 (1.24∼2.03)	13.451	<0.001
Allergic disease (yes vs. no)	–0.273	0.76 (0.57∼1.02)	3.416	0.065	–0.673	0.51 (0.31∼0.84)	6.896	0.009
Eosinophils	–0.419	0.66 (0.44∼0.98)	4.148	0.042	–0.864	0.42 (0.21∼0.84)	6.038	0.014
Lymphocyte ratio	–0.035	0.97 (0.96∼0.98)	42.047	<0.001	–0.073	0.93 (0.91∼0.95)	71.185	<0.001
HDL	0.606	1.83 (1.38∼2.43)	17.768	<0.001	0.932	2.54 (1.70∼3.79)	20.647	<0.001
Apolipoprotein A1	–0.581	0.56 (0.38∼0.82)	9.123	0.003	–0.796	0.45 (0.26∼0.79)	7.888	0.005
Plasma fibrinogen	0.079	1.08 (1.03∼1.14)	9.359	0.002	0.092	1.10 (1.02∼1.18)	6.268	0.012
Fructosamine	0.114	1.12 (1.04∼1.20)	10.035	0.002	0.170	1.18 (1.07∼1.31)	10.276	0.001
Oxygen partial pressure	0.001	1.00 (1.00∼1.00)	1.788	0.181	0.004	1.00 (1.00∼1.01)	7.267	0.007
Plasma carbon dioxide concentration	0.004	1.00 (1.00∼1.01)	2.785	0.095	0.014	1.01 (1.01∼1.02)	15.666	<0.001
Bicarbonate standard value	0.089	1.09 (1.05∼1.13)	23.390	<0.001	0.120	1.13 (1.07∼1.18)	22.675	<0.001
Partial pressure of carbon dioxide	0.085	1.09 (1.07∼1.10)	159.799	<0.001	0.148	1.16 (1.14∼1.18)	290.662	<0.001

Serum iron, magnesium, ESR, CEA, myoglobin, serum procalcitonin, residual base in extracellular fluid, base excess, percentage of naive granulocytes, absolute value of naive granulocytes, hsCRP, and D-dimer were removed giving more than 1,000 missing value.

### Prediction model

A prediction model was constructed based on the results of multivariate analysis (Table 4). The model correctly predicted COPD severity in 60.17% of cases (kappa coefficient: 0.35; 95% CI: 0.33–0.37). The model’s sensitivity for mild/moderate, severe, and very severe COPD was 72.31, 56.28, and 40.71%, respectively, whereas the specificity for these categories was 72.94, 66.07, and 94.99%, respectively. The accuracy for these categories was 72.68, 61.96, and 85.69%, respectively.

**TABLE 4:** Evaluation of the multinomial logit prediction model of chronic obstructive pulmonary disease (COPD) severity.

Predictive performance	Mild/Moderate	Severe	Very severe	Total	
Mild/moderate	1,319	652	61	2,032	Correctly predicted: 60.17%
Severe	486	1,053	392	1,931	Kappa: 0.35 (0.33∼0.37)
Very severe	19	166	311	496	
Total	1,824	1,871	764	4,459	
Sensitivity	72.31%	56.28%	40.71%		
Specificity	72.94%	66.07%	94.99%		
Accuracy	72.68%	61.96%	85.69%		

## Discussion

None of the existing prediction models for COPD severity assessed patient prognosis using biochemical and immunological parameters (13–15). After adjusting for potential confounders, we found that the parameters that could distinguish between mild/moderate and severe cases were vascular disorders, HDL, plasma fibrinogen, fructosamine, standard bicarbonate concentration, pCO_2_, age, eosinophil count, lymphocyte ratio, and apolipoprotein A1. The factors that could distinguish between mild/moderate and very severe cases were vascular disorders, HDL, plasma fibrinogen, fructosamine, pO_2_, plasma carbon dioxide concentration, standard bicarbonate concentration, pCO_2_, age, sex, allergic diseases, eosinophil count, lymphocyte ratio, and apolipoprotein A1. The prediction model correctly predicted disease severity in 60.17% of the cases, with a kappa coefficient of 0.35.

Several studies have developed prediction models for COPD severity based on various parameters (14, 17). For instance, Chen et al. developed a model for predicting disease severity in patients hospitalized for COPD exacerbation and found that neutrophil count percentage and demographic parameters were associated with a higher risk of COPD exacerbation; the area under the receiver operating characteristic curve was 0.84 (14). Pertzov et al. used capnography to predict obstruction severity in non-intubated patients with COPD and asthma using a prediction model containing several waveform features, age, sex, and height (17). However, no study has developed a prediction model based on biochemical and immunological parameters in hospitalized patients with stable COPD.

The predictors of COPD severity were vascular disorders, HDL, plasma fibrinogen, fructosamine, standard bicarbonate concentration, pCO_2_, age, eosinophil count, lymphocyte ratio, apolipoprotein A1, pO_2_, plasma carbon dioxide concentration, sex, and allergic diseases. This result may be due to several reasons: (1) systematic inflammatory responses could explain the increased risk of severity of COPD in patients with vascular disorders (18); (2) apolipoprotein M binds to HDL, which is significantly associated with inflammatory factors, including serum and lung platelet-activating factor and leptin levels (19–21); (3) the levels of plasma fibrinogen, an acute phase reactant synthesized in hepatocytes, are significantly correlated with COPD severity and exacerbation risk (22, 23); (4) fructosamine could reflect average blood glucose concentration over 2–3 weeks, and elevated fructosamine could reflect hyperglycemia state (24); (5) blood bicarbonate is associated with acid-base disorders, which are significantly related to COPD severity (25); (6) pCO_2_ is significantly related to higher scores for disease severity indicators (BODE or GOLD) and is a good predictor of severe COPD (26); (7) the association of age and sex with the risk of severe COPD could be explained by selection bias because we used a first admission sample and all patients were recruited from a single center; (8) eosinophilia is associated with lower dyspnea scores, reduced functional impairment, and better response to inhaled corticosteroids, potentially reducing COPD severity (27); (9) lymphocyte ratio is related to immune ability; and (10) fractional exhaled nitric oxide is associated with COPD severity and allergic airway inflammation (28).

Our model had a moderate ability to differentiate COPD severity based on the selected variables and correctly predicted severity in 60.17% of the patients. The predictive performance of this model was better than that of the null model. Moreover, the sensitivity and specificity for detecting mild/moderate cases were 72.31 and 72.94%, and accuracy was 72.68%. The sensitivity and specificity for detecting severe cases were 56.28 and 66.07%, and accuracy was 61.96%. The sensitivity and specificity for detecting very severe disease were 40.71 and 94.99%, and accuracy was 85.69%. This result suggests that the model’s ability to differentiate severe COPD was low, while the accuracy for detecting very severe COPD (85.69%) and mild/moderate COPD (72.68%) was high.

This study firstly constructed a prediction model based on biochemical and immunological parameters in hospitalized patients with stable COPD. This prediction model could used to assess the severity of COPD for patients are unable to perform breath test, and the ratings COPD severity could generate automatically based on electronic medical record. Thus, high risk patients could identified using constructed model, and early treatment strategies could provide to improve the prognosis of COPD.

Several strengths of this study should be highlighted: (1) the current retrospective cohort study contained large sample size, and the conclusion was robustness; (2) both univariate and multivariate analyses were applied to identify potential predictive factors; (3) the prediction model was constructed and a risk scoring system was established based on multivariate analyses; and (4) the predictive value of constructed model was assessed using sensitivity, specificity, accuracy, and Cohen’s kappa test.

This study has limitations. First, the retrospective design may lead to selection and recall bias. Second, non-treatment may have increased COPD severity. Third, serum iron, magnesium, ESR, CEA, myoglobin, serum procalcitonin, extracellular fluid volume depletion, base excess, the percentage and absolute number of granulocytes, hsCRP, and D-dimer were excluded from the multivariate analysis because of missing data. Fourth, the prediction model was not validated externally.

## Conclusion

This study identified predictors of COPD severity, and a prediction model was constructed using biochemical and immunological parameters from patients from a single center. The predictors of COPD severity included vascular disorders, HDL, plasma fibrinogen, fructosamine, standard bicarbonate concentration, pCO_2_, age, eosinophil count, lymphocyte ratio, apolipoprotein A1, pO_2_, plasma carbon dioxide concentration, sex, and allergic diseases. The model’s ability to predict very severe COPD was high. Nonetheless, larger studies are needed to validate these findings.

## Data availability statement

The raw data supporting the conclusions of this article will be made available by the authors, without undue reservation.

## Ethics statement

The studies involving human participants were reviewed and approved by Suining Central Hospital. The ethics committee waived the requirement of written informed consent for participation.

## Author contributions

Y-FG: conceptualization, data curation, methodology, supervision, writing—original draft, project administration, and writing—review and editing. Y-FG and LC: data curation and writing—review and editing. LC: methodology, formal analysis, software, writing—review and editing, and investigation. Y-FG and RQ: methodology and writing—review and editing. S-HW and PC: investigation, methodology, and writing—review and editing. All authors contributed to the article and approved the submitted version.
